# The Telephone as an Alcoholometer

**Published:** 1904-12

**Authors:** 


### THE TELEPHONE AS AN ALCOHOLOMETER.

It is stated that M. Meneuvrier, assistant director of the Laboratory of Researches of the Paris Faculty of Sciences, has just discovered an infallible method of ascertaining by the use of the telephone how much a given quantity of wine has been watered. The principle on which the invention rests is the variable conductivity of different liquids.

The apparatus is described as follows: Two vessels, one containing wine known to be pure, the other the same quantity of wine to be tested, are placed on an instrument outwardly resembling a pair of scales. The telephone is in contact with both liquids. If the sample of wine under observation is as pure as the standard used for comparison no sound is heard; if, on the contrary, it contains water, the tell-tale telephone "speaks," and the greater the proportion of water the louder the instrument complains. A dial on which a number of figures are marked is connected with the telephone.

To ascertain the proportion of water in the wine tested, the operator moves a hand on the dial until the telephone, which has been "speaking" all this time, relapses into silence. The hand has thus been brought to a certain figure on the dial. This number is then looked up in a chart which the inventor has drawn up and corresponding to it is found indicated the exact proportion of water contained in the quantity of wine.

M. Meneuvrier's remarkable invention can, he says, be easily applied to the testing of many other liquids and even solids, which may be adulterated by the addition of foreign matter possessing a conductivity different from that of the original substance.—*The Medical Times.*

